# Ultrasound-Assisted Extraction of Syringin from the Bark of *Ilex rotunda* Thumb Using Response Surface Methodology

**DOI:** 10.3390/ijms13067607

**Published:** 2012-06-20

**Authors:** Li-Chun Zhao, Ying He, Xin Deng, Xiang-Hua Xia, Jian Liang, Geng-Liang Yang, Wei Li, Hui Wang

**Affiliations:** 1 College of Pharmacy, Hebei University, Baoding 071002, China; E-Mails: hyzlc@126.com (L.-C.Z.); xhxiagx@126.com (X.-H.X.); 2 The Affiliated Ruikang Hospital, Guangxi University of Chinese Medicine, Nanning 530011, China; E-Mails: hyinggx@yeah.net (Y.H.); dx2000@126.com (X.D.); ljruikang@126.com (J.L.); 3 College of Chinese Medicinal Materials, Jilin Agricultural University, Changchun 130118, China; E-Mail: liwei7727@126.com; 4 China-Japan Union Hospital, Jilin University, Changchun 130033, China

**Keywords:** ultrasound-assisted extraction, syringin, *Ilex rotunda*, response surface methodology, Box-Behnken design

## Abstract

In this work, a rapid extraction method based on ultrasound-assisted extraction (UAE) of syringin from the bark of *Ilex rotunda* Thumb using response surface methodology (RSM) is described. The syringin was analyzed and quantified by high performance liquid chromatography coupled with UV detection (HPLC-UV). The extraction solvent, extraction temperature and extraction time, the three main factors for UAE, were optimized with Box-Behnken design (BBD) to obtain the highest extraction efficiency. The optimal conditions were the use of a sonication frequency of 40 kHz, 65% methanol as the solvent, an extraction time of 30 min and an extraction temperature of 40 °C. Using these optimal conditions, the experimental values agreed closely with the predicted values. The analysis of variance (ANOVA) indicated a high goodness of model fit and the success of the RSM method for optimizing syringin extraction from the bark of *I. rotunda*.

## 1. Introduction

The bark of *Ilex rotunda* Thumb, a traditional Chinese herbal drug (Jiubiying in Chinese), belongs to the Aquifoliaceae family. It is used for the treatment of colds, tonsillitis, sore throat, acute gastroenteritis, and dysentery in China [1]. The main bioactive constituents of *I. rotunda* are triterpenoid saponins [2], phenolic acid compounds [3] and flavanoids. In addition, syringin as an important kind of phenyl-propanoid glycoside is widely regarded to be an important component of *I. rotunda* [4]. Recently, syringin has received increasing attention because of various biological activities such as anti-inflammation [5,6] neuroprotective effect [7] and anti-hyperglycemic activity [8–10].

**Figure 1:**
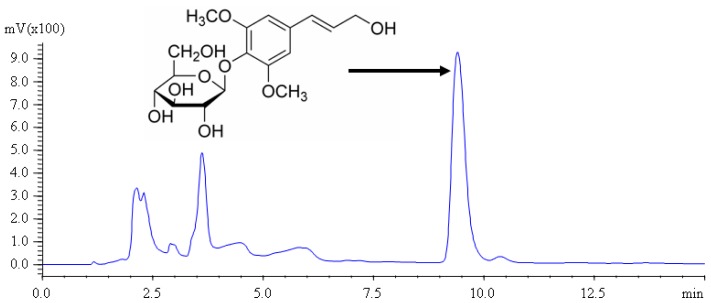
The structure and HPLC chromatogram of syringin in the bark of *I. rotunda*.

In the last years, efficient and fast extraction methods, such as ultrasound-assisted extraction (UAE), have been employed as alternative extraction methods for the natural constituents from plant material [11–16]. The UAE technique is attractive because of its simplicity and low equipment cost. Furthermore, it is based on the employment of the energy derived from ultrasounds to facilitate the extraction of analytes from the solid sample by the organic solvent [17,18]. Generally, the UAE method with conventional ultrasonic cleaners is considered to better extract natural products [19]. As has been shown, many factors including ultrasound power, extraction time, extraction temperature, and solvent to solid ratio affected the extraction efficiency of the UAE method [20]. The conventional optimization methods usually investigate one variable at-a-time, which is not only time consuming, but also fails to consider the possible interactions between different variables. Response surface methodology (RSM), an effective statistical technique, can optimize complex extraction procedures by investigating the variables and the interactions of the variables simultaneously [21,22]. Figure 1 showed the structure of syringin.

Though UAE technique is a novel method to effectively extract total flavonoids from *I. rotunda* [23], there are no reports on the extraction of syringin *I. rotunda* using the UAE method. In this paper, response surface methodology was applied to optimize ultrasound-assisted extraction of syringin from *I. rotunda* for the first time. Several important factors, such as extraction solvent, extraction time and extraction temperature, were systemically analyzed using a Box-Behnken design combined with response surface methodology.

## 2. Results and Discussion

### 2.1. Model Fitting

In the present investigation, we selected three main parameters for optimization of the extraction of the syringin from *I. rotunda*. Furthermore, preliminary trials were carried out in order to establish a more realistic extraction mode. Finally, the range of methanol concentrations (30–100%), extraction time (10–50 min) and extraction temperature (20–80 °C) was fixed. Table 1 presents the experimental design and corresponding response data for the extraction of syringin.

Table 2 shows the analysis of variance (ANOVA) for the extraction yield of syringin from *I. rotunda* using Box-Behnken design. The determination coefficient (*R*^2^) of the model is 0.9880, with no significant lack of fit at *p* > 0.05. That means that the calculated model was able to explain 99.80% of results. The results indicated that the model used to fit response variable was significant (*p* < 0.0001) and adequate to represent the relationship between the responses and the independent variables [24]. The *F*-value, 64.22, implied that the model was highly significant. The adjusted determination coefficient (*R*^2^_adj_) of 0.9726 indicated that only 2.74% of the total variations can not be explained by the calculated model. Meanwhile, the coefficient of variation (C.V.% = 1.59) indicated that the model was reproducible.

Table 3 shows that syringin extraction yield was affected significantly by all three variables (*p* < 0.001). It was evident that all the quadratic parameters (*X*_1_^2^, *X*_2_^2^, *X*_3_^2^) and one interaction parameter (*X*_1_*X*_2_) were significant at the level of *p* < 0.0001, whereas the other interaction parameters (*X*_1_*X*_3_, *X*_2_*X*_3_) were insignificant (*p* > 0.1). The predicted second-order polynomial model was:

$$Y=9.15-0.031X_{1}+0.065X_{2}-0.034X_{3}-0.18X_{1}X_{3}+0.047X_{2}X_{3}-1.31X_{1}^{2}-0.67X_{2}^{2}-0.053X_{3}^{2}$$

where *Y* is the yield of syringin (mg/g), and *X*_1_, *X*_2_ and *X*_3_ are the coded variables for methanol concentration, extraction time and extraction temperature, respectively.

### 2.2. Response Surface Optimization of UAE Condition

**Figure 2:**
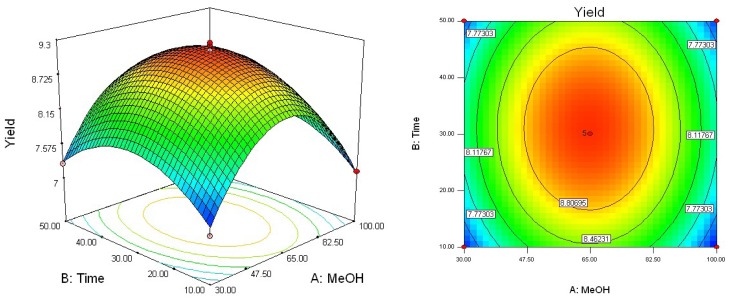
Response surface plot of methanol concentration and extraction time.

**Figure 3:**
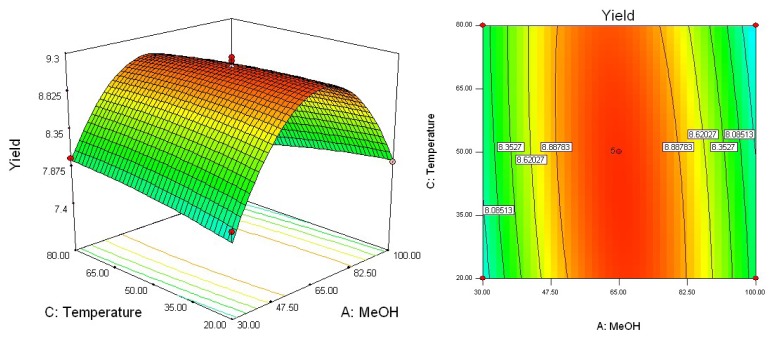
Response surface plot of methanol concentration and extraction temperature.

**Figure 4:**
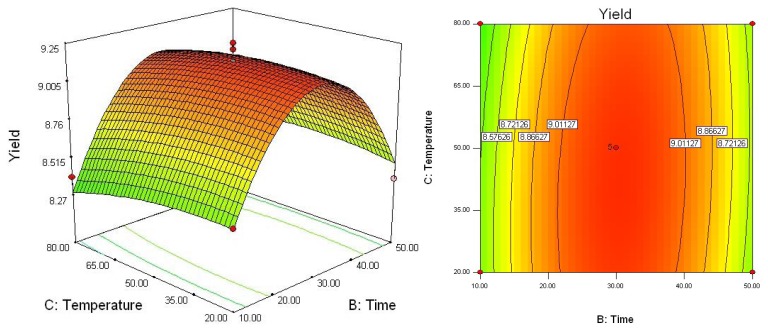
Response surface plot of extraction temperature and extraction time.

To determine optimal levels of the variables for the syringin extraction, the three-dimensional surface plots were constructed according to equation. Figures 2–4 show the effects of the independent variables and their mutual interaction on the extraction yield of syringin in *I. rotunda*.

Figure 2 shows the effect of methanol concentration (*X*_1_) and extraction time (*X*_2_) on the yield of syringin in *I. rotunda* at a fixed extraction temperature. At a definite extraction time, the extraction yield increased slightly with methanol concentration from 30 to 65% and nearly reached a peak at the highest extraction time. However, upon increasing the methanol concentration beyond 65%, there was a gradual decline in the response, and extraction times over 30 min did not show any obvious effect on extraction yield. The results may be explained by the fact that increasing extraction time may accelerate chemical decomposition of syringin due to durative ultrasonic effect, which leads to the lower extraction yield [25]. Although ultrasound-assisted extraction has considerable potential in extraction fields of natural constituents, the flavor and composition of some edible oils is deteriorated after ultrasound treatment [26,27]. Hence, it is necessary to investigate the effect of ultrasound on other constituents and antioxidant of *I. rotunda* in further investigation.

Figure 3 shows the effect of methanol concentration (*X*_1_) and extraction temperature (*X*_3_) on the yield of syringin in *I. rotunda*. Upon increasing the methanol concentration from 30 to 65% with an increase of extraction temperature from 20 to near 50 °C, the extraction yield of syringin increased with methanol concentration. The results are in accord with the data in Table 3, which showed that the interactive effect of methanol concentration with extraction temperature on the yield of syringin was not very weak (*p* > 0.001).

Figure 4 shows the effect of the interaction of extraction time (*X*_2_) and extraction temperature (*X*_3_) on the extraction yield. The results indicate that the highest extraction yield could be achieved when using 50 °C as the extraction temperature and 30 min as the extraction time. However, the extraction yield gradually decreased with extraction temperatures over 50 °C. It could be explained that, as extraction temperature increased, more impurities were extracted, resulting in a lower overall yield of syringin [26].

### 2.3. Optimization of Extraction Parameters and Validation of the Model

In the present investigation, the software predicted that the optimum methanol concentration, extraction time and extraction temperature were 65.35%, 30.74 min and 40.39 °C, respectively. The software predicted the optimized extraction yield of syringin to be 9.16 mg/g. Table 4 shows three parallel experiments which were carried out under the optimal conditions, in which the average extraction yield of syringin was 9.20 mg/g. Compared with the value predicted by Design-Expert 7.1.6, the results show that the predicted value was very close to the actual result, indicating that the optimization parameters proposed are reliable.

### 2.4. Comparison with Conventional Extractions

As indicated in the introduction, conventional extraction methods were used for sample preparation of syringin in *I. rotunda*. Hence, it is necessary to compare the extraction yield of syringin when UAE is used compared to conventional extraction methods. In the present investigation, merceration extraction (ME) was employed under the same extraction conditions as UAE. The results indicated that the yield of syringin when ME was used (6.22 mg/g) was far lower than when UAE was used (9.20 mg/g). UAE has absolute advantage when compared to ME for the extraction yield of syringin.

## 3. Experimental Section

### 3.1. Plant Material

The dried bark of *I. rotunda* was bought from a drug store in Guangxi Province and identified by Dr. Li-Chun Zhao. The cut pieces were ground to obtain a relatively homogenous powder (0.2–0.5 mm). The powder was dried at 60 °C to a constant weight and was well blended before use.

### 3.2. Chemicals and Reagents

HPLC grade methanol was purchased Fisher Chemicals (USA). Other chemicals, such as ethanol and methanol, were all of analytical grade from Beijing Chemical Factory (Beijing, China). Water was purified using a Milli-Q water purification system (Millipore, USA). Standard of syringin was purchased from the National Institute for the Control of Pharmaceutical and Biological Products of China (Beijing, China).

### 3.3. Ultrasound-Assisted Extraction

In the present study, an ultrasonic cleaning bath (KQ-250DB, Kunshan Electronics Co., Ltd., China) with a frequency of 40 kHz and a maximum peak power of 250 W was employed to investigate UAE extraction of syringin. About 1.0 g of sample was placed in the ultrasonic bath and sonicated at 40 kHz for a certain time at different extraction temperatures. The filtrate was collected and the residue was extracted again (two times) with the same volume of fresh solvent. According to preliminary trials (data not shown), some experimental parameters, *i.e*., methanol as extraction solvent, 80 mesh material size, 1/20 solid to solvent ratio and 40 kHz of sonication working frequency, were suitable for the process. All the samples were prepared and analyzed in triplicate.

### 3.4. Merceration Extraction

Merceration extraction (ME) as one of the conventional extractions was performed according to the optimal condition determined with UAE. In brief, 1.0 g drug powder of *I. rotunda* was extracted with 65% methanol as extraction solvent, an extraction temperature of 40 °C of and extraction time of 30 min. This process was repeated for three cycles.

### 3.5. HPLC Analysis of Syringin

The syringin in *I. rotunda* was quantified by high performance liquid chromatography coupled with UV detection (HPLC-UV). The analysis was performed with a HPLC instrument (Agilent 1100, USA) equipped with a quaternary solvent delivery system, a column oven and UV detector. Separation was achieved on a Hypersil ODS2 column (4.6 mm × 250 mm, 5 μm) from Dalian Elite Analytical Instruments Co., Ltd. (Dalian, China). The column temperature was set at 25 °C and detection wavelength was set at 265 nm. The mobile phase was 10% CH_3_CN with a flow rate of 1.0 mL/min. The isocratic elution was employed with 20 μL of injection sample.

### 3.6. Experimental Design

Box-Behnken design (Design-Expert software, Trial Version 7.1.6, Stat-Ease Inc., Minneapolis, MN, USA) was applied to determine the best combination of extraction variables for the yield of syringin in *I. rotunda*. Three extraction variables considered in this investigation were *X*_1_ (methanol concentration), *X*_2_ (extraction time) and *X*_3_ (extraction temperature). The proper range of the three variables was determined according to single-factor experiments. The whole design consisted of 17 experimental points as listed in Table 1, and five replicates (run 13–17) at the center of the design were used for estimating a pure error sum of squares.

### 3.7. Data Analysis

Data were expressed as standard errors of the means (SEM) of three replicated determinations. The response obtained from each set of experimental design was subjected to multiple non-linear regressions using the Design-Expert software. The quality of the fit of the polynomial model equation expressed by the coefficient was checked by *F*-test and *p*-value.

## 4. Conclusions

In the present paper, the ultrasound-assisted extraction of syringin from *I. rotunda* was performed with a three-variable, three-level Box-Behnken design (BBD) based on response surface methodology (RSM). The experimental results showed that all three factors contributed to the extraction of syringin. As such, it may be said that UAE is an effective and indeed feasible method for the extraction of syringin from the bark of *I. rotunda*.

## Figures and Tables

**Table 1:** Box-Behnken experimental design with the independent variables.

Run	Variables levels			Extraction yield (mg/g)

	*X*_1_, methanol (%)	*X*_2_, time (min)	*X*_3_, temperature (°C)	
1	30.00	10.00	50.00	7.01
2	100.00	10.00	50.00	7.11
3	30.00	50.00	50.00	7.25
4	100.00	50.00	50.00	7.34
5	30.00	30.00	20.00	7.81
6	100.00	30.00	20.00	7.94
7	30.00	30.00	80.00	7.99
8	100.00	30.00	80.00	7.42
9	65.00	10.00	20.00	8.45
10	65.00	50.00	20.00	8.38
11	65.00	10.00	80.00	8.39
12	65.00	50.00	80.00	8.51
13	65.00	30.00	50.00	9.05
14	65.00	30.00	50.00	9.11
15	65.00	30.00	50.00	9.13
16	65.00	30.00	50.00	9.21
17	65.00	30.00	50.00	9.25

**Table 2:** Analysis of variance for the fitted quadratic polynomial model of extraction of syringin.

Source	Sum of squares	Degree of freedom	Mean square	*F*-value	Prob > F	
Model	9.77	9	1.09	64.22	< 0.0001	significant
Residual	0.12	7	0.02			
Lack of fit	0.093	3	0.03	4.83	0.0812	not significant
Pure error	0.026	4	< 0.001			

**Table 3:** Estimated regression model of the relationship between response variables (extraction yield of syringin) and independent variables (*X*_1_, *X*_2_, *X*_3_).

Variables	Degree of freedom	Sum of squares	*F*-values	*p*-value
*X*_1_	1	0.0078	0.46	0.5184
*X*_2_	1	0.0338	2.00	0.2002
*X*_3_	1	0.0091	0.54	0.4866
*X*_1_*X*_2_	1	<0.001	<0.01	0.9704
*X*_1_*X*_3_	1	0.1225	7.25	0.0310
*X*_2_*X*_3_	1	0.0090	0.53	0.4886
*X*_1_^2^	1	7.1981	426.01	<0.0001
*X*_2_^2^	1	1.8620	110.20	<0.0001
*X*_3_^2^	1	0.0116	0.69	0.4346

**Table 4:** Optimum conditions and the predicted and experimental yield at the optimum conditions.

	Methanol (%)	Extraction time (min)	Temperature (°C)	Yield of syringin
Optimum conditions	65.35	30.74	40.39	9.16 (predicted)
Modified conditions	65	30	40	9.20 (actual)
